# Sex differences in disease severity and immune responses in murine and human inflammatory arthritis

**DOI:** 10.1186/s13293-026-00840-w

**Published:** 2026-02-07

**Authors:** Mahadevappa Hemshekhar, Liam J O’Neil, Nyambura Kahia, Courtney L. Marshall, Tamarah Singh, Mario Navarrete, Hani El-Gabalawy, Neeloffer Mookherjee, Janilyn Arsenio

**Affiliations:** 1 Department of Internal Medicine, Rady Faculty of Health Sciences, University of Manitoba, Winnipeg, MB Canada; 2 Nursing Department, School of Health and Community Services, Red River College Polytechnic, Winnipeg, MB Canada; 3 Manitoba Centre for Proteomics and Systems Biology, Winnipeg, MB Canada; 4 Department of Immunology, Rady Faculty of Health Sciences, University of Manitoba, Winnipeg, MB Canada

**Keywords:** Collagen-induced arthritis, Rheumatoid arthritis, Sex differences, Immune responses

## Abstract

**Background:**

Rheumatoid Arthritis (RA), a systemic autoimmune disorder of unknown etiology, disproportionately affects females at a 3:1 ratio compared to males. While biological sex differences in the immune system exist, sex-related differences in inflammatory and immune mediators of RA disease severity are undefined. Our objective was to characterize sex-related differences in immune responses in a murine collagen-induced arthritis (CIA) model and in human RA patients.

**Methods:**

In CIA compared to saline control mice, inflammatory disease severity was assessed using standardized clinical scores. Anti-collagen antibodies, neutrophil elastase, calprotectin/ S100A8/A9 heterodimer, CRAMP, MMP3, and MMP9 were quantified by ELISA in the sera and joint tissues. Cytokine/chemokine levels in sera and joints were assessed using a Luminex based-44-Plex Discovery Assay^®^ Array. Immunophenotyping of mouse splenic T cells analysis was performed by flow cytometry. Proteomic profiling of serum samples from an established RA cohort (72 female and 19 male that were at least 84% ACPA+) was performed using an aptamer-based SomaScan platform.

**Results:**

We identified distinct sex-related differences in disease severity and pro-inflammatory profiles in the sera and joint tissues of CIA mice, with inflammatory responses that were male-skewed in the sera and female-skewed in the joints. Furthermore, we demonstrated heightened neutrophil activation markers and CD4^+^ T cell-mediated inflammatory responses in female CIA mice. Similar sex-related differences in neutrophil activation and leucocyte migration were identified in RA patients.

**Conclusions:**

Our study demonstrates novel sex differences in pro-inflammatory mediators and activities of neutrophils and CD4^+^ T cells associated with disease severity in CIA mice, and in human RA patients. These findings provide new insights into sex-related differences in immunological pathways associated with inflammatory arthritis, which may contribute to the sex disparity in RA pathogenesis.

**Supplementary Information:**

The online version contains supplementary material available at 10.1186/s13293-026-00840-w.

## Abstract (plain language summary)

Rheumatoid arthritis is a disease where the immune system attacks its own tissues and leads to pain and swelling, also known as inflammation, of the joints. It is known that rheumatoid arthritis affects females more than males. A better understanding of how the immune system responds to inflammatory arthritis between females and males may help to uncover female- and male-associated factors that may contribute to the course of the disease. This study assessed immune responses to inflammatory arthritis in female and male mice and in rheumatoid arthritis patients. We discovered that in mice that developed inflammatory arthritis, males had greater paw and joint swelling compared to females. When we measured immune responses in diseased mice, although male mice showed elevated levels of inflammatory molecules in the serum, these markers were higher in the joints of female diseased mice. Our study further found that the activities of two types of immune cells, called neutrophils and T cells, were biased towards females in both diseased mice and in human rheumatoid arthritis patients. These results indicate that immune responses to inflammatory arthritis work differently in females and males, which may affect sex differences in disease severity. This research may help future studies uncover mechanisms underlying sex differences in arthritis development and sex specific therapeutic targets. (212 words)

**Supplementary Information:**

The online version contains supplementary material available at 10.1186/s13293-026-00840-w.

## Highlights

- Using a murine collagen-induced arthritis (CIA) model, female and male CIA mice develop disease symptoms (e.g. joint swelling, inflammation) compared to their saline control groups.
- Male CIA mice exhibit greater disease severity than female CIA mice.
- CIA mice show sex differences in inflammatory responses in the serum and joint tissues.
- Elevated neutrophil activation markers and CD4^+^ T cell-mediated responses are female-biased in CIA mice and in Rheumatoid Arthritis patients.

**Supplementary Information:**

The online version contains supplementary material available at 10.1186/s13293-026-00840-w.

## Introduction

Rheumatoid Arthritis (RA), a common chronic and systemic autoimmune disorder, is primarily characterized by inflammation of the synovial joints, leading to pain, swelling, and progressive joint destruction [1]. RA is a multifactorial disease where a combination of genetic predisposition, environmental triggers (e.g. smoking, microbial infections), and immunological dysregulation contribute to the loss of immune tolerance [2, 3]. RA affects approximately 1% of the global population, with a significant sex disparity in prevalence, symptoms, severity, and response to therapy [4]. RA impacts females at a ratio of 3:1 compared to males, although some studies suggest males can develop more severe erosive disease [1, 4]. The combinatorial impact of sex steroids [5], genetic factors [3, 6], X-linked expression of miRNAs and other immunomodulatory genes associated with RA, environmental exposures, and other factors, play a multifactorial role in the sex-related differences that manifest in RA [3–6]. Despite the prominent role of inflammatory response and immune dysregulation in RA, the immunological changes and associated cellular mechanisms underlying sex differences in RA remain unclear [5, 7].

Both innate and adaptive immune systems play critical roles in RA onset and pathogenesis [8, 9]. Myeloid cells secrete pro-inflammatory cytokines and chemokines to drive inflammation and activate adaptive autoreactive T and B cells [9, 10]. Synovial macrophages, fibroblasts, and neutrophils produce pro-inflammatory cytokines to perpetuate inflammation [8, 11]. In particular, neutrophils are primed to secrete increased amounts of reactive oxygen species (ROS) and a repertoire of inflammatory cytokines and chemokines in the RA joints [8, 10, 11]. Consequently, neutrophil degranulation can mediate tissue damage in the joints by secreting ROS, and other enzymes such as neutrophil elastase (NE) [11]. Neutrophils also bridge innate and adaptive immune responses by promoting T cell migration and differentiation [10], and activating autoreactive B cells [12]. Emerging evidence suggests that the interactions between neutrophils and T cells are critical in shaping the immune landscape of RA [10]. Formation of neutrophil extracellular traps (NETs) facilitates the citrullination of specific proteins, which in turn leads to B cell production of anti-citrullinated protein antibodies (ACPAs), a hallmark of RA [11]. Thus, B cells contribute to RA pathogenesis by producing ACPA autoantibodies, in addition to rheumatoid factor (RF) [11]. ACPA antibody responses are augmented by CXCR5 expressing follicular CD4^+^ T cells (Tfh) [10, 13]. In addition to CD4^+^ Tfh cells, other T cell subsets contribute to RA pathogenesis, including Th17 cells, regulatory CD4^+^ T cells (Treg) [14], and cytotoxic CD8^+^ T cells [15]. The interplay of the different immune cell types highlights the complexity of the immune network in RA pathogenesis. It is known that innate and adaptive immune responses are qualitatively and quantitatively different between females and males [5, 7]. Investigations over the last decade have enhanced our understanding of sex-related differences in arthritis. However, limited studies have examined sex differences in immune cell composition and functions in the pathogenesis of inflammatory arthritis in mice and RA in humans [16].

Inflammatory arthritis animal models, particularly the collagen-induced arthritis (CIA) murine model [17], enable investigations into the pathophysiology of RA. CIA is one of the most widely used experimental models for RA as it closely mimics the clinical, immunological, and histopathological features of the human RA [17]. However, previous studies showed inconsistencies in the appearance of clinical symptoms in the CIA model [18, 19]. Second, while a few studies have reported a sex disparity in the disease severity in CIA mice [18, 20], primarily male mice have been used, as female mice showed weaker disease induction in the CIA model [18, 19]. To overcome these challenges, we modified the CIA model to synchronize the disease onset [21–23]. Here, we use the synchronized CIA model, which shows comparable clinical disease onset in both female and male mice, to interrogate the influence of sex as a biological variable in immune and inflammatory responses which may be associated with disease severity.

In this study, we identified sex-related differences in inflammatory arthritis using a murine model of CIA and in a well-established RA cohort that was mostly ACPA positive, > 80% positive in both sexes. In the CIA model, although the clinical disease severity and erosive markers were higher in males, inflammatory responses associated with degranulating neutrophils and activated CD4^+^ T cells were significantly higher in females compared to males. Similar to our findings in the CIA model, we found that cytokine biosignatures related to neutrophil degranulation and CD4^+^ T cell activation were higher in female compared to male human RA patients. Altogether, these findings define specific sex-related differences in cytokine biosignature and immune pathways that may contribute to sex dimorphism in the disease severity, and thereby differences in treatment responses in inflammatory arthritis.

## Methods

### Synchronized collagen-induced arthritis (CIA) murine model

All animal work was approved by the Central Animal Care Committee of the University of Manitoba. Experimental design and reporting of data are compliant with the ARRIVE guidelines for in vivo animal research [24]. Utilization of the CIA murine model is described in our previous studies [21–23]. Briefly, male and female DBA/1J mice (∼ 6 weeks old) were obtained from The Jackson Laboratory, acclimatized for at least 2 weeks for housing and environment considerations, and received a standard diet, prior to collagen challenge. Mice were anesthetized using isoflurane (3.5%) and challenged with a subcutaneous (s.c.) injection of 100 µg bovine collagen type II (CII) emulsified in complete Freund’s adjuvant in a total volume of 100 µl in the tail. A boost of 50 µg CII emulsified in incomplete adjuvant (total volume of 50 µl) was administered s.c. in the tail on day 21 after the initial CII challenge. Mice were administered with LPS from E. coli 0111:B4 (20 µg per mouse) intraperitoneally (i.p.) on day 25 after the first CII challenge. All reagents for the CIA challenge were obtained from Chondrex Inc. (WA, USA). Saline was administered s.c. in the tail to the collagen-naïve mice on day 1 (100 µl) and day 21 (50 µl) [21, 22]. CIA challenge was performed between 10 am and 1 pm for all mice. Mice were visually monitored for grooming and activity levels daily. Disease severity was assessed by monitoring joint thickness using a digital caliper everyday starting from day 22 after the first CII challenge. Disease severity was assessed using a standardized clinical score based on joint thickness, in a blinded manner, as previously described by us [21, 22], and a clinical score ranging from 0 to 16 was assigned to each mouse by summing the scores of each limb. On day 29 after the first CII challenge, mice were anesthetized with isoflurane and euthanized by cardiac puncture. Blood by cardiac puncture for serum and joints were collected. Serum was aliquoted and stored at −20 °C and joint tissues were flash frozen and stored at −80 °C, until use.

### Serum anti-collagen type II (CII) antibodies

Serum levels of mouse anti-collagen antibodies (autoantibodies) and bovine anti-collagen antibodies (antibodies to the immunizing antigen) were determined by ELISA using a Mouse Anti-mouse Type II Collagen IgG Antibody Assay Kit and Mouse Anti-Bovine Type II Collagen IgG Antibody Assay Kit, respectively, according to the manufacture’s protocol (Chondrex Inc. WA, USA). The antibody concentrations in the test samples were calculated by comparison with the optical density (OD) values of standard anti-CII antibody (units/ml) [21, 22].

### Joint tissue homogenization

Joint tissue homogenates were prepared for examining the abundance of cytokines, as previously described by us [21, 22]. Briefly, mice joints collected were cleaned to remove skin/tissues, flash frozen in liquid nitrogen and stored at −80 °C until use. The flash frozen joint tissue samples were homogenised on ice in Protein Extraction Reagent T-Per (Thermo Fisher Scientific, IL, USA) containing protease inhibitor cocktail (PIC; Cell Signalling Technology, MA, USA) using the Cole-Parmer LabGEN 125 homogenizer (Cole-Parmer Canada, QC, Canada). For determining NE activity, joint tissue homogenates were prepared without PIC. Tissue homogenates were centrifuged at 12,000×*g* for 15 min at 4 °C and supernatants were collected. Total protein concentration was determined in the tissue lysate supernatants using Bicinchoninic acid (BCA) assay (Thermo Fisher Scientific). The lysates were aliquoted and stored at −80 °C until use [21, 23].

### Cytokine/chemokine quantification in mouse serum and joint tissue

A panel of 44 cytokines and chemokines were quantified in serum and joint tissue lysates using the Mouse Cytokine/Chemokine 44-Plex Discovery Assay^®^ Array (MD44), a Luminex-based assay platform (Eve Technologies Corporation, Calgary, AB). The 44 analytes measured are detailed in Supplementary Tables 1 and 2. Fluorescent intensities of samples that were below the lowest standard concentration or undetected were imputed using half the value of the lowest standard concentration for the respective analyte.

### Quantification of selected proteins in mice serum and joint tissue

The abundance of NE (R&D Systems Quantikine kit, Catalog # MELA20), calprotectin i.e. the S100A8/A9 heterodimer (R&D Systems Duo set, Catalog # DY8596-05), mouse cathelicidin peptide CRAMP (Biomatik, Catalog # EKC36669) and matrix metalloproteinases MMP3 (R&D Systems Duo set, Catalog # DY548) and MMP9 (R&D Systems Duo set, Catalog # DY6718), were quantified in mice serum and joint tissue lysates using specific enzyme-linked immunosorbent assay (ELISA) kits. For each ELISA, joint tissue lysates used contained 50 µg of total protein and serum samples were diluted either 1:10 or 1:20 as required. Each ELISA was performed according to the manufacturer’s instructions.

### Neutrophil elastase (NE) activity in joint tissue lysates

NE activity was measured by adding 1:25 diluted joint tissue lysates to a reaction mixture containing 2.5 µM of fluorescence-quenching substrate (Peptide institute, Cat # 3232-v) in 100 mM HEPES buffer (pH 7.4) containing 750 mM NaCl and 0.05% IGEPAL CA-630 (non-ionic detergent) [25]. The reaction was monitored at 37 °C for 20 min with fluorescent acquisition readings each minute at Ex/Em 320/420 nm in a microplate reader Synergy H1 (Agilent technologies, Inc).

### Antibodies and flow cytometry analysis of mouse splenic T cells

Antibodies (BioLegend) included: CD4 (RM4-5), CD8α (53 − 6.7), CD62L (MEL-14), CD44 (1M7), IFN-γ (XMG1.2), (IL-17) (TC11-18H10.1), and IL-22 (Poly5164). For intracellular staining of IFN-γ, IL-17, and IL-22, CIA and saline control splenocytes were cultured ex vivo with a cell Stimulation Cocktail (500X) of phorbol 12-myristate 13-acetate and ionomycin (ThermoFisher Scientific) in the presence of brefeldin A (Sigma) for 6 h at 37 °C. For intracellular cytokine detection, cells were stained with surface antibodies then fixed in 4% paraformaldehyde (Electron Microscopy Services) and permeabilized before staining with intracellular antibodies. All samples were acquired on an Accuri C6, FACSAria II (BD Biosciences) or CytoFLEX (Beckman Coulter), and data analyzed using FlowJo V10 software.

### Analysis of serum proteome from a RA cohort

RA patient serum was collected and stored from an established longitudinal study which sought to recruit First Degree Relatives of RA patients and RA probands. Serum proteins from 90 RA patients were quantified using SomaScan (kit version 1.3), a proteomics assay that measures ~ 1,300 proteins using an aptamer library according to the manufacturer’s instructions. Proteins are expressed in fluorescence units, which were log_2_ transformed prior to analysis.

### Statistical analysis

GraphPad Prism 10 software was used for data analyses. As indicated in the Figure legends, statistical significance was determined by One-way analysis of variance (ANOVA) followed by Tukey multiple comparison *post hoc* test or Fisher’s LSD test to determine the statistical significance between the groups. A *p*-value of ≤0.05 was considered to be statistically significant. The heatmaps and volcano plots were generated using R (v4.4.2). SomaScan data were analysed in R (v4.4.2) using a custom pipeline with packages for data analysis and visualization (ggplot2, ConsensusclusterPlus, dplyr). Gene ontology enrichment and gene set enrichment analysis was undertaken using clusterProfiler.

## Results

### Disease severity is significantly higher in male compared to female CIA mice

**Fig. 1:**
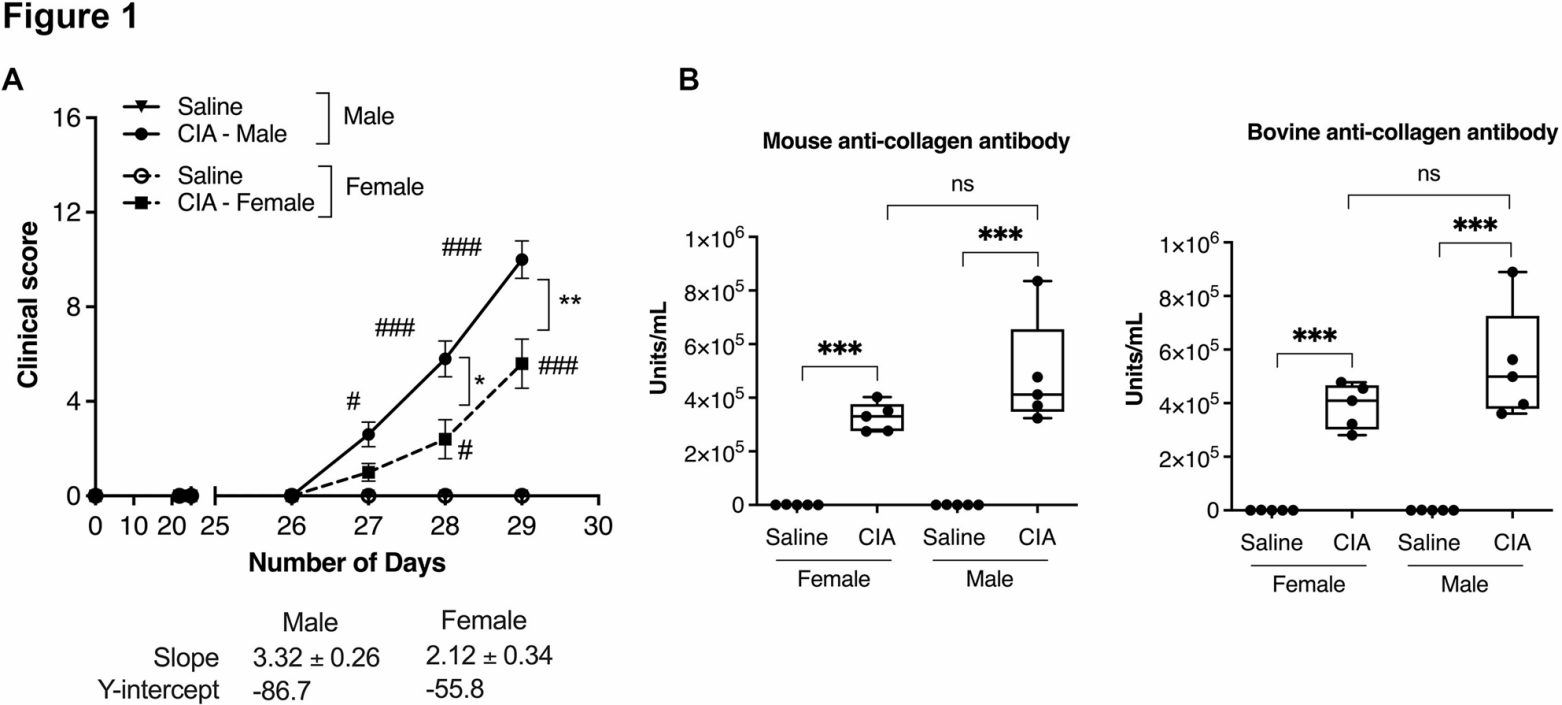
Male CIA mice exhibit higher disease severity compared to females. Male and female CIA and saline control mice were monitored for disease severity and assigned clinical scores from day 21 after the first CII challenge until the end of experiment (day 29). **(A)** Line graphs representing the mean clinical scores of mice starting from day 1 to day 29. On day 29 after the first CII challenge, mice were euthanized by cardiac puncture under anesthesia for blood and serum collection and storage at −80 °C. Serum concentrations of **(B)** anti-mouse collagen type II antibodies (left panel) and anti-bovine collagen type II antibodies (right panel) were analyzed by ELISA. *N* = 5 mice per group. One of two independent experiments. Simple linear regression analysis was performed to determine the statistical difference between the lines. One-way analysis of variance (ANOVA) followed by Tukey’s multiple comparison *post hoc* test was used to determine the statistical significance between the groups. (*/^#^*p* ≤ 0.05, **p ≤ 0.005 and ***/^###^*p* ≤ 0.0005). In Fig. 1A, ^#^significance of comparisons between CIA and saline control mice; *significance of comparisons between sexes of CIA mice

To monitor the disease severity, we measured the clinical scores, assigned based on joint thickness as described in the methods [21, 22]. Both male and female CIA mice developed disease symptoms such as joint swelling and inflammation compared to their respective saline control group (Fig. 1A). However, male CIA mice showed a significantly higher paw and joint swelling, resulting in higher clinical scores (> 40%) compared to female CIA mice (Fig. 1A). The slope of the line corresponding to mean values of clinical scores from day 26 to day 29 showed that male CIA mice had the higher value for the slope of trend line representing severity of the symptoms (3.32 ± 0.26), compared to female CIA mice with values of (2.12 ± 0.34) **(**Fig. 1A**)**. These results indicated that the female CIA mice exhibit lower severity and a delayed disease onset compared to male CIA mice.

### Anti-collagen type II antibodies are significantly elevated in both male and female CIA mice

Immunization with bovine CII in DBA/1J mice induces the production of antibodies against both the bovine CII and mouse CII autoantigen [21, 22]. Therefore, we measured both bovine CII and murine CII antibodies in the serum using ELISA [21, 22]. Contrary to the clinical scores, serum levels of both murine CII (Fig. 1B left panel) and bovine CII (Fig. 1B right panel) were significantly elevated in both male (median ~ 400, 000 and ~ 500, 000 Units/mL, respectively) and female CIA mice (median ~ 300, 000 and ~ 400, 000 Units/mL, respectively) compared to their respective saline control groups. Although no statistical sex differences were observed in the abundance of CII antibodies, the median of both murine CII and bovine CII were marginally higher in male CIA mice (by ~ 100, 000 Units/mL) compared to females (Fig. 1B).

### Upregulation of inflammatory cytokine/chemokine profile is female-biased in joint tissues and male-biased in the serum

**Fig. 2:**
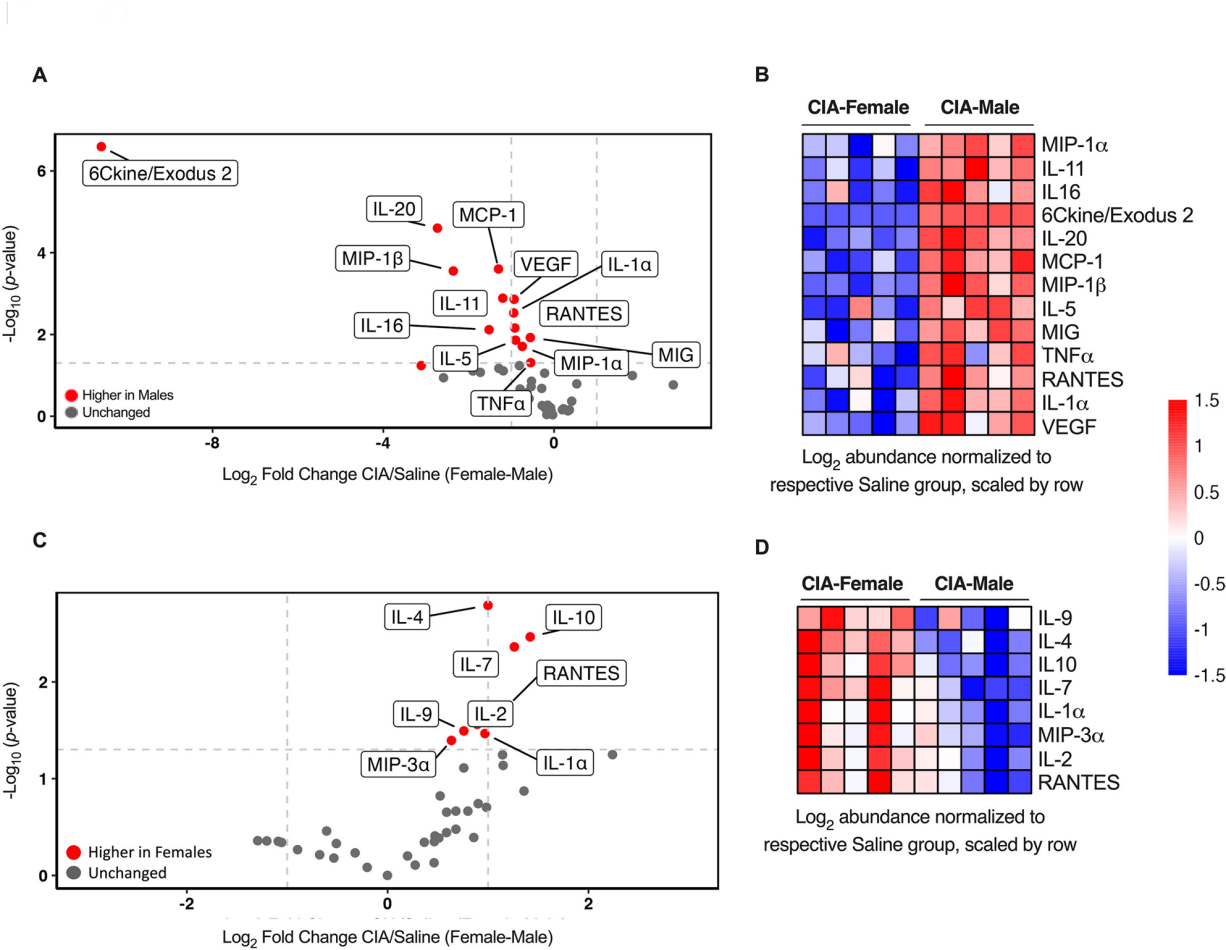
Upregulated cytokine/chemokines profiles show a male bias in serum and female bias in joint tissues. On day 29 after the first CII challenge, mice were euthanized. Serum and joint tissue lysates were collected. A panel of 44 cytokines and chemokines were evaluated in the serum and joint tissue lysate samples using mouse cytokine/chemokine 44-plex discovery assay array (MD44). The volcano plot analysis of **(A)** serum and **(C)** joint tissue was performed with Log_2_ fold-change (CIA-Saline) values. The red dots indicate higher in females, blue dots indicate higher in males and gray dots indicate unchanged values. The heatmap analysis performed with Log_2_ fold-change (CIA-Saline) values on the selected (based on the volcano plot analysis) cytokine/chemokine in **(B)** serum **(D)** joint tissue showed the differential abundance of cytokine and chemokines in male and female mice. Cytokine and chemokine profiles of CIA mice were represented as Log_2_ values normalized to respective saline group with spread across the scale of − 1.5 to + 1.5-fold (blue to red). *N* = 5 mice per group. One of two independent experiments

Cytokines and chemokines that are secreted by immune cells, such as T cells, neutrophils and macrophages, play a critical role in the pathogenesis of RA by mediating the inflammatory response and driving the autoimmune processes that lead to joint damage [8, 10, 11]. Therefore, we evaluated a panel of 44 cytokines and chemokines in the mice sera and joint tissues using the Luminex-based multiplex platform. The list of 44 cytokines/chemokines demonstrating the magnitude of change with corresponding *p*-values in both serum and joint tissue are listed in Supplementary Tables 1 and 2. We found striking sex-related differences in cytokine/chemokine profiles in the serum (Fig. 2A and B) and joint tissue (Fig. 2C and D), in CIA mice compared to saline groups. The volcano plots and heatmap analysis demonstrated significant differences in the cytokine/chemokine profile between male and female CIA mice (Fig. 2A and D). Interestingly, a sex-related difference in the cytokine profiles was found between serum and joint tissues. The serum profile of cytokine/chemokines in male CIA mice was higher compared to female CIA mice (Fig. 2A and B), whereas female CIA mice exhibited higher abundance of cytokines/chemokines in the joint tissues compared to males (Fig. 2C and D). These results demonstrated that the inflammatory cytokines/chemokine profile significantly elevated in CIA mice compared to saline control mice was female-biased in joint tissues and, in contrast, male-biased in the serum.

### Joint inflammation and degradation markers are higher in male compared to female CIA mice

**Fig. 3:**
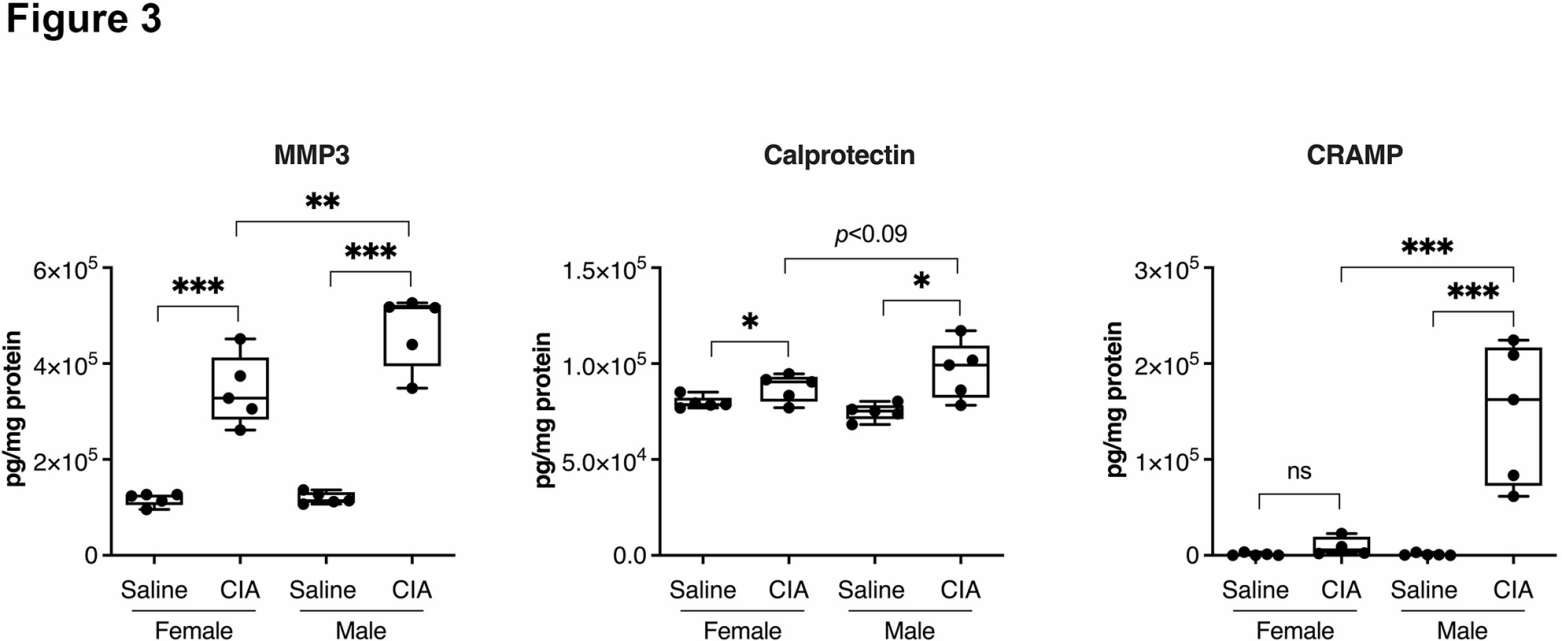
Inflammation and degradation associated proteins are elevated in male compared to female CIA joint tissues. On day 29 after the first CII challenge, mice were euthanized and joint tissues were collected. CRAMP (left panel), Calprotectin (S100A8/A9) (middle panel), and MMP3 (right panel) were quantified by ELISA (according to manufacturer’s protocol) in joint tissue lysates of CIA and saline control mice. 50 µg of protein was used from the joint lysates to perform each ELISA. Statistical analysis was performed using One-way ANOVA followed by Fisher’s LSD test to determine the statistical significance (*p ≤ 0.05 and **p ≤ 0.01, ***p ≤ 0.001). *N* = 5 mice per group. One of two independent experiments

We have previously demonstrated that the antimicrobial host defence peptides such as cathelicidin CRAMP and calprotectin (S100A8/A9), and enzymes such as matrix metalloproteinase-3 (MMP3) are significantly elevated in the CIA mice joints [23, 26]. Therefore, we measured these peptides and proteins in the mice joints using ELISA. Calprotectin and MMP3 levels were significantly elevated in both male and female CIA mice compared to their respective saline controls (Fig. 3), whereas, CRAMP, in particular, was highly elevated in male, but not female CIA mice (Fig. 3). Overall, sex-related differences were observed, with male CIA mice joints showing significantly higher levels of CRAMP (> 2-fold), S100A8/A9 (> 0.5-fold), and MMP3 (> 1-fold) compared to female CIA mice (Fig. 3). Our results demonstrated that although the inflammatory cytokine/chemokine profile is higher in the joints of female CIA mice (Fig. 2), joint inflammation and degradation associated markers such as CRAMP, calprotectin and MMP3 are higher in male CIA mice. These results align with those of previous comparative studies wherein severe pain and joint erosion in male compared to female RA patients were observed [27, 28].

### Neutrophil elastase enzyme abundance and activity are higher in female CIA mice

**Fig. 4:**
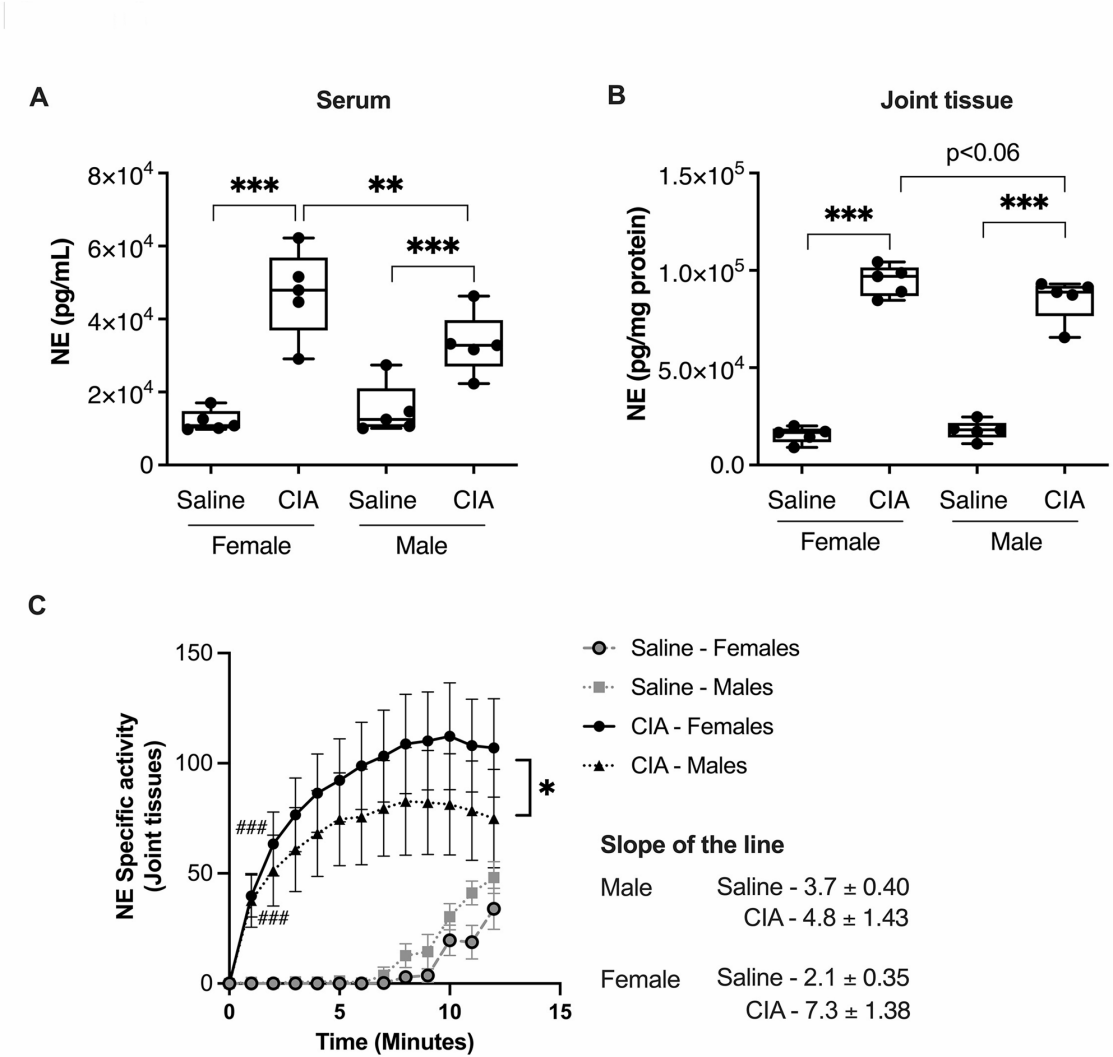
Neutrophil elastase (NE) abundance and enzyme activity are higher in female compared to male CIA mice. On day 29 after the first CII challenge, mice were euthanized, and serum and joint tissues were collected. **(A)** The abundance of NE in both serum and joint tissue lysates of CIA and saline control mice was determined using ELISA, according to the manufacturer’s protocol. 50 µg of protein was used from the joint lysates to perform the ELISA. **(B)** The NE enzyme activity in the joint tissue lysate was determined using a fluorescent substrate-based activity assay in CIA compared to saline control mice. Simple linear regression analysis was performed to determine the statistical difference between the lines. One-way ANOVA followed by Fisher’s LSD test to determine the statistical significance between the groups (*p ≤ 0.05 and **p ≤ 0.01, ***/^###^*p* ≤ 0.001). In Fig. 4C, ^#^significant compared to saline-control; *significant compared to male and female CIA group. *N* = 5 mice per group. One of two independent experiments

Neutrophil elastase (NE) is a serine protease secreted by activated neutrophils and plays a key role in the pathogenesis of RA [11]. NE contributes to tissue destruction and inflammation by degrading extracellular matrix components, amplifying inflammation by generating pro-inflammatory peptides and cytokines, and triggering the formation of neutrophil extracellular traps (NETs). We demonstrated that NE abundance was elevated in both the serum and joint tissues of male and female CIA mice, with NE enzymatic activity significantly increased in the joints compared to their respective saline controls. However, both NE abundance (> 2-fold) and activity (> 1-fold) were significantly higher in the serum and joint tissue of female compared to male CIA mice, indicating a sex-related difference in NE regulation (Fig. 4A and C). This data suggests that although disease severity is higher in male CIA mice, the innate immune activity could be higher in female CIA mice.

### Activated CD4^+^ T cells are higher in female CIA mice, whereas as pro-inflammatory cytokine producing CD4^+^ T cell subsets are higher in males

**Fig. 5:**
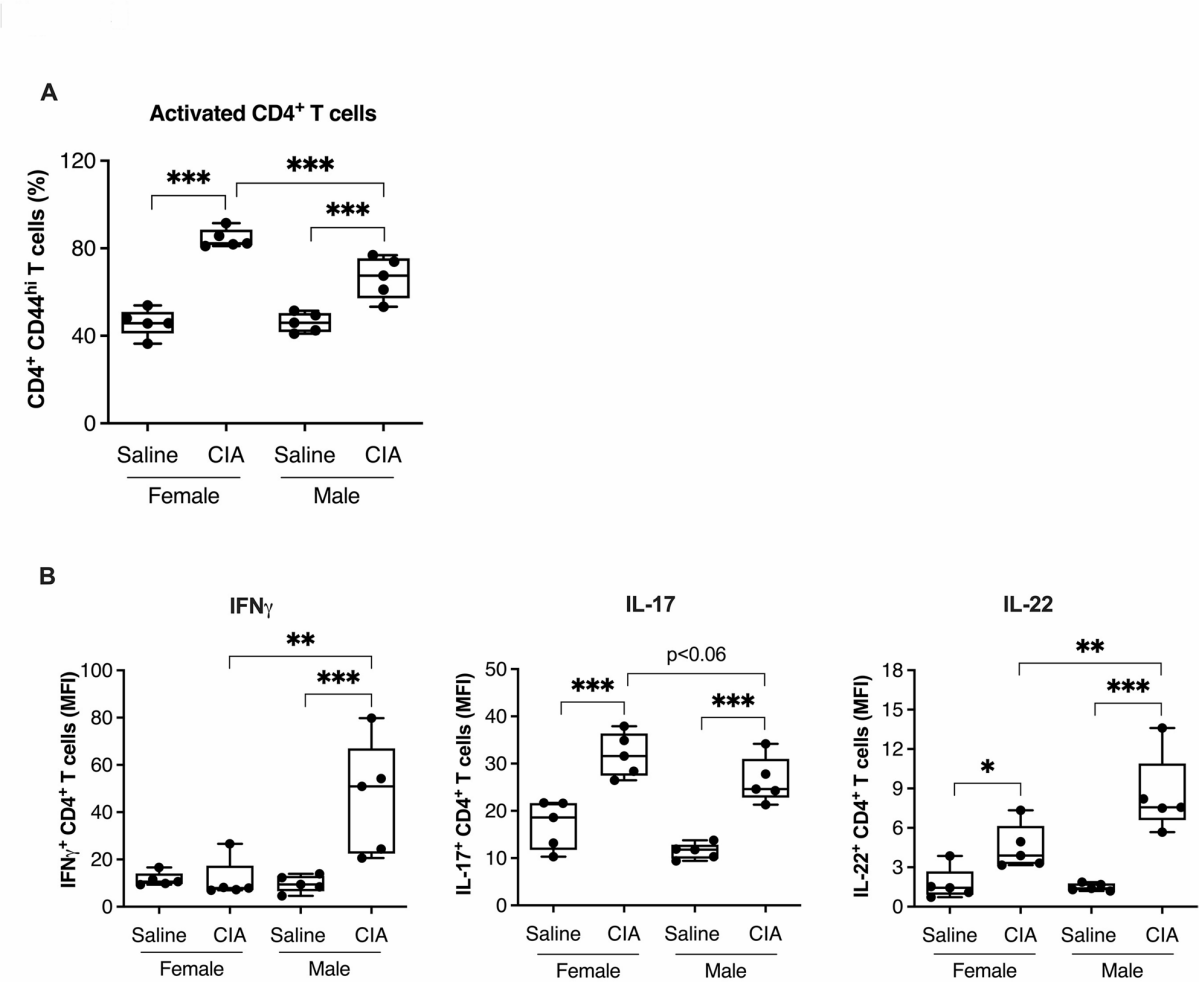
Sex-related differences in activated CD4^+^ T cells in CIA compared to saline control mice. Flow cytometry analysis of splenic T cells from CIA and saline control mice day 29 after the first CII challenge. Frequency (%) of **(A)** activated CD4^+^ T cells (CD44^hi^), and **(B)** mean fluorescence intensity (MFI) of intracellular IFNγ (left panel), IL-17 (middle panel), and IL-22 (right panel) expression of activated CD4^+^ T cells in CIA compared to saline control female and male mice. Statistical analysis was performed using One-way ANOVA followed by Fisher’s LSD test to determine the statistical significance (*p ≤ 0.05 and **p ≤ 0.01, ***p ≤ 0.001). *N* = 5 mice per group. One of two independent experiments

CD4^+^ T cells are the predominant lymphocytic infiltrate in the synovitis of RA patients [29]. We next evaluated the accumulation of T cell subsets in the spleens of CIA compared to saline control mice using flow cytometry. Our data showed that both male and female CIA mice exhibited a significant increase (> 0.75–1.0.75.0-fold) in the frequency of activated (CD44^hi^) [30, 31] CD4^+^ T cells compared to their respective saline control group (Fig. 5A). Interestingly, the frequency of activated CD4^+^ T cells was significantly higher in the female compared to male CIA mice (Fig. 5A). Ex-vivo stimulation of splenic T cells with PMA (Phorbol 12-myristate 13-acetate) and ionomycin in the presence of brefeldin A demonstrated significantly higher mean fluorescence intensities (MFI) (>2-fold increase) of intracellular expression of inflammatory cytokines IFNγ, IL-17, and IL-22 in activated CD4^+^ T cells in male compared to female CIA mice (Fig. 5B). This data demonstrates that CD4^+^ T cell-mediated inflammatory responses are strikingly distinct in male and female CIA mice.

### Serum proteomic profile indicates neutrophil activation in female but not male RA patients

**Fig. 6:**
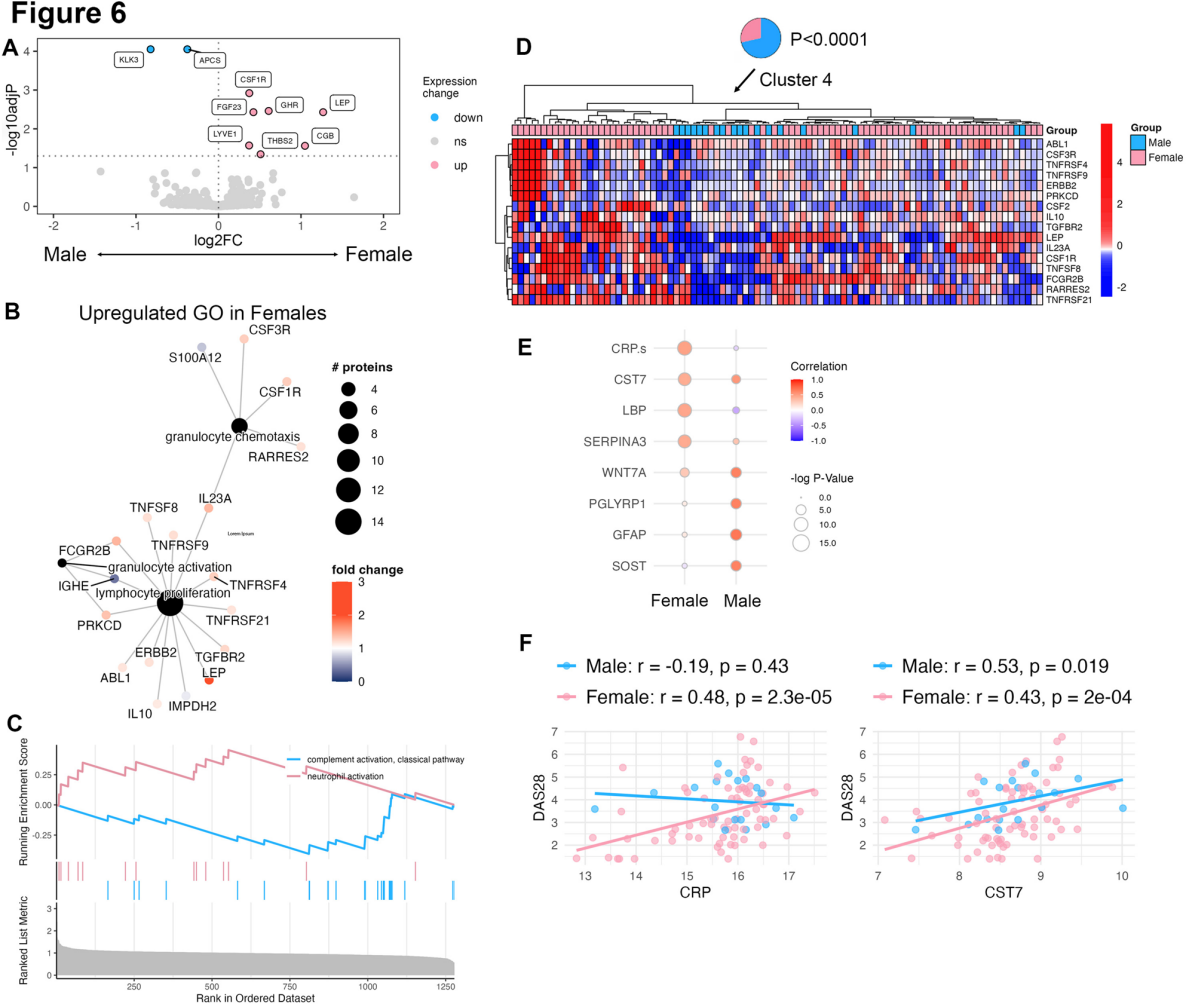
Neutrophil activation is enriched in female, but not in male RA. **(A)** Differentially expressed protein between female and male RA patients (*n* = 92). **(B)** Upregulated Gene Ontology (GO) pathways in female RA, most suggestive of leukocyte proliferation and granulocyte chemotaxis. **(C)** Gene Set Enrichment Analysis (GSEA) distinguishes female RA by neutrophil activation and male RA by complement activation. **(D)** Hierarchical clustering of patient samples based on GO pathways suggests revealed 5 distinct clusters. Cluster 4 was enriched in male RA patients (52% of total, *p* < 0.0001). **(E)** Correlation between serum proteins and DAS28 (*N* = 4 for each sex shown) reveals little overlap between proteins that correlate strongly with DAS28 between each sex. **(F)** Spearman correlation for CRP and DAS28 is discordant between sexes, while CST7 is strongly associated with DAS28 in both sexes

The clinical characteristics of the RA patient cohort can be found in Supplementary Table 3, split by sex (72 female, 19 male). The RA cohort was mostly ACPA positive (84.5% female and 89.5% male) with a mean Disease Activity Score-28 (DAS28) that was higher in males compared to females (3.9 vs. 3.4, *p* = 0.04). We quantified 1305 serum proteins using SomaScan and undertook principle component analysis (PCA), which revealed minimal clustering based on sex (Supplementary Fig. 1A) or disease activity measured by DAS28, based on achieving low disease activity/remission status as recommended by treatment guidelines in RA [32] (Supplementary Fig. 1B). Next, we performed a differential expression analysis between RA females and males. After adjusting for multiple comparisons, we identified 7 proteins that were upregulated in female patients, and 2 proteins that were upregulated in male patients (Fig. 6A). The highly upregulated proteins in females included colony stimulating factor-1 receptor (CSF1R) and fibroblast growth factor-23 (FGF23), while highly upregulated in males were serum amyloid P component (APCS) and serine protease, kallikrein related peptidase 3 (KLK3) (Supplementary Fig. 2). Gene Ontology (GO) analysis revealed an enrichment of 318 pathways (Supplementary Fig. 3) including *leukocyte proliferation* and *granulocyte chemotaxis* (Fig. 6B). Furthermore, Gene Set Enrichment Analysis (GSEA) revealed several enriched gene sets which included *granulocyte activation* (*p* = 0.007) in females and *complement activation* (*p* < 0.0001) in males, suggestive of distinct inflammatory pathways that are sex specific (Fig. 6C). Using proteins in the GO pathways, we performed hierarchical clustering on the RA samples, revealing distinct clusters of patients based on protein expression of these markers. Indeed, most of the male patients (52%) and only 5% of the female patients were assigned to Cluster 4 (*p* < 0.0001) suggesting that a subset of the human proteome enriched in leukocyte/neutrophil proteins are highly expressed in females and can distinguish RA patients based on sex (Fig. 6D). We further calculated a neutrophil activation protein score, which was significantly higher in female (*p* = 0.004) compared to male RA patients (Supplementary Fig. 4) further suggesting that the distinguishing feature of the RA proteome in females is a neutrophil activation signature.

To determine if there are sex-related differences in serum proteins related to disease activity, we calculated the spearman correlation between the individual serum proteins and DAS28 within each sex. 44 proteins (*R* > 0.4, *p* < 0.05) significantly correlated with DAS28 in males, while 4 proteins were significantly correlated in females. Interestingly, only 2 proteins, Cystatin F (CTS7) (*R* = 0.53 in males, *R* = 0.42 in females) and Wnt family member 7 A (WNT7A) (*R* = 0.63 in males, 0.28 in females) were significantly associated with DAS28 in both sexes **(**Fig. 6E**)**. As others have shown, C-reactive protein (CRP) varies substantially based on sex [33]. In our data, CRP was strongly associated with female DAS28, but this was not observed in our male cohort **(**Fig. 6F**)**, suggesting that additional markers may provide value above CRP in the assessment of RA disease activity. Overall, this data suggests that the underlying immunological drivers in RA disease activity varies substantially in each sex, with a neutrophil signature underpinning female RA.

## Discussion

In this study, we sought to gain new insights into sex-related differences in inflammatory arthritis. Using a murine model of synchronized CIA, we demonstrate that the clinical symptoms are more severe in CIA males with increased erosive disease markers in the joints and inflammatory cytokines in the serum, compared to females. We found that female CIA disease is skewed towards degranulating neutrophils and activated CD4^+^ T cells, with an increased inflammatory cytokine profile in the joints, compared to males. Similarly, in the cohort of RA patients, we found an increased abundance of inflammatory and neutrophil activation markers in female compared to male RA patients. Altogether, our data suggest that sex-related differences in immune responses are associated with the severity of inflammatory arthritis in CIA mice and in human RA patients.

Biological sex plays a key role in the prevalence, severity, and response to therapy in RA [5, 7, 34]. Although RA affects approximately 1% of the global population, females are impacted at a ratio of 3:1 compared to males [2, 4, 7, 34]. This sex disparity underscores the influence of hormonal, genetic, and immune-related factors in disease susceptibility [2, 4, 7, 34]. A majority of immunomodulatory-related genes are encoded on the X-chromosome, which further contributes to amplified immune responses in females in chronic diseases, increasing their predisposition to autoimmunity [3, 16, 35]. Additionally, females and males differ in their immune cell repertoires and elicit different immune responses during infection and chronic diseases. In general, females exhibit more robust adaptive immune responses compared to males, which may contribute to a differential regulation of inflammation in RA [16, 35].

The sex disparity in disease symptoms and severity of RA remains unelucidated. Therefore, in this study, we first aimed to characterize sex related differences in disease symptoms and severity using a CIA model. We found that male CIA mice exhibited more severe disease symptoms compared to female CIA mice and have increased levels of anti-collagen antibodies. An increase in clinical scores, circulating inflammatory cytokine/chemokine profiles in the serum, and joint degradation markers were male biased. Pro-inflammatory cytokines/chemokines such as 6Ckine/Exodus 2 (CCL21), MCP-1 (CCL2), IL-20, IL-16, IL-11, TNFα, IL-1α, RANTES (CCL5), MIP-1α (CCL3), and MIG (CXCL9) were significantly higher in circulation of male compared female CIA mice. These proinflammatory molecules can orchestrate the persistent immune dysregulation in RA leading to chronic inflammation and tissue damage. For example, cytokines such as TNF-α and IL-1α can amplify synovial inflammation, promoting fibroblast-like synoviocyte (FLS) activation and matrix degradation [36]. Similarly, cytokine IL-20 can induce osteoclastogenesis and bone erosion [37]. Chemokines such as 6Ckine/Exodus 2, MCP-1, RANTES, MIP-1α, and MIG and IL-16 can recruit and activate T cells and monocytes, fueling immune cell infiltration [38]. Previous studies have demonstrated that prevalence of clinical arthritis in CIA mice is similar in females and males but with different kinetics, wherein onset of clinical features of inflammatory arthritis and prevalence is delayed in females compared to male CIA [18]. Thus, it is possible that clinical scores, circulating inflammatory cytokine profile and joint degradation markers in female CIA mice may be similar to that observed in males at a later time point of outcome assessments, in the synchronized CIA murine model used in this study which warrants further investigation.

On the other hand, an elevated cytokine/chemokine profile in the joint tissues was female biased. Interestingly, we found that both proinflammatory cytokines and chemokines, such as IL-7, IL-2, IL-9, RANTES, MIP-3α, and IL-1α, as well as anti-inflammatory cytokines, such as IL-10 and IL-4, were higher in female compared to male CIA joints. Low levels of serum cytokines/chemokines and increased levels of anti-inflammatory cytokines in the joints of female CIA mice may explain their lower clinical scores compared to their male counterparts, despite increased proinflammatory cytokines in the female CIA mice joints. It is known that inflammatory markers are enhanced in individuals at-risk of RA prior to the onset of clinical symptoms [39, 40]. It is thus likely that the enhanced inflammatory cytokine profile in the joints of female CIA mice represents changes in biological processes that drive the clinical onset of inflammatory arthritis, which needs to be further investigated. Sex-related differences in molecular networks and biological processes in the preclinical phase of inflammatory arthritis i.e. before the onset of clinical symptoms has not yet been defined.

The clear male bias in disease severity observed in our study is in line with previous studies that demonstrated male mice have a higher susceptibility to develop arthritis and disease severity [41, 42], which, in part, could be attributed to the capacity of female sex hormones to suppress the development of CIA [41, 42]. Sex hormones such as estrogen, progesterone, and androgens can modulate the immune system, particularly in the context of RA. While we did not measure sex steroid levels in our study, several studies have demonstrated that estrogen and progesterone levels play key roles in defining the susceptibility and severity of RA disease [41–43]. The declining levels of estrogen and progesterone are linked to the increased risk of RA development [44, 45]. For example, decreased levels of estrogen and progesterone during menopause and postpartum increases the risk of RA, whereas increased estrogen and progesterone during pregnancy are protective against RA [44]. Studies using overiectomized DBA/1J mice and SKG mice reported increased inflammatory arthritis disease symptoms with increased levels of TNFα and IL-6 compared to sham operated mice [46, 47]. Interestingly, exogenous administration of estrogen and progesterone significantly reduced the disease severity and the serum levels of TNFα and IL-6. In addition, estrogen receptor alpha (ERα) was shown to mediate estrogen-driven suppression of disease symptoms in rat adjuvant arthritis [41, 42]. These studies highlight the critical role of sex hormones in arthritis development [46, 47] and provide an explanation for the male bias in disease severity observed in our study.

Although female CIA mice showed less disease severity compared to their male counterparts, we found that female CIA disease is skewed towards degranulating neutrophils and activated CD4^+^ T cells. It is well documented that RA onset and severity involves a dynamic interplay between innate and adaptive immune responses [10, 29]. Particularly, neutrophils and T cells play interdependent and critical roles driving both acute and chronic inflammatory processes that lead to joint damage [10, 29]. Neutrophils in the synovial fluid secrete proteases including NE, MMPs, and CRAMP that degrade cartilage and bone [10, 11, 48]. Further, secretion of NETs not only enhances inflammation, but exposes citrullinated proteins, key autoantigens implicated in the production of ACPAs, a classical feature of RA [10, 11, 48]. NETs have been shown to play an important role in both CIA and RA. A reduction in CIA disease severity has been reported in PAD4^-/-^ deficient mice or with pharmacological inhibition of PAD4, which is highly specific for NET formation [49, 50]. Whether this observation is due to citrullinated antigens or non-antigen functions of NETs, such as stimulating stromal and immune cells, or proteolytic cleavage of cartilage and other joint structures, remains to be determined. In parallel, CD4^+^ T cells, particularly Th1 and Th17 subsets, dominate the adaptive immune landscape in RA [10, 14, 51, 52]. CD4^+^ Th1 cells secrete IFN-γ, which activates macrophages to release pro-inflammatory cytokines such as TNFα and IL-1, while CD4^+^ Th17 cells produce IL-17, a potent mediator of neutrophil recruitment and osteoclast activation [10, 51, 53]. The interplay between neutrophils and CD4^+^ T cells is critical: neutrophil-derived NETs expose autoantigens which amplify T cell activation and proliferation, and T cell-mediated production of proinflammatory cytokines such as IL-17, enhances neutrophil infiltration and activation [10]. This self-sustaining loop underscores the complexity of RA pathogenesis. Our findings of CIA male-biased increased levels of CRAMP, and calprotectin in the joints, and IFNγ, IL-17 and IL-22 expressed by CD4^+^ T cells; and CIA female-biased increased CD4^+^ T cells and neutrophil activation markers such as NE, highlight the potential that different inflammatory and immunological mechanisms underlie disease severity differences in male versus female CIA mice.

In addition, in RA patients, despite higher disease activity in males (DAS28, *p* < 0.05), the female serum proteome profiles demonstrated enrichment of proteins in granulocyte and neutrophil activation determined by GO enrichment and GSEA. We speculate that, in females, neutrophil and CD4^+^ T cell activities switch to regulatory processes [10, 29, 54–56]. NE can tightly regulate immune NETosis, where the degradation of NETs can reduce autoantigen exposure and subsequent autoantibody production, potentially dampening the disease response [57, 58]. Additionally, NE may degrade specific pro-inflammatory cytokines/chemokines in a controlled manner, limiting synovial inflammation [54, 55]. NE can also remodel extracellular matrix components to release bioactive fragments with regulatory properties [54, 55]. Regulatory CD4^+^ T cells (Tregs) can control inflammation in RA by inhibiting the activation of Th1 and Th17 cells, thereby reducing IFN-γ and IL-17 production [9, 59]. Tregs can also induce the secretion of anti-inflammatory cytokines such as IL-10 and transforming growth factor-beta (TGF-β) in the synovium, reducing joint inflammation and damage. Future studies will investigate whether sex differences in diverse T cell subsets such as Tregs are associated with RA disease onset and severity. Altogether, our results suggest that neutrophil and CD4^+^ T cells may be programmed to regulate the disease in female mice and RA patients, in contrast to amplifying immune responses in males.

In summary, our study reveals novel sex-related differences in pro-inflammatory mediators and activities of neutrophils and CD4^+^ T cells, in CIA mice and in human RA patients. The findings of this study provide the foundation for future studies aimed at deciphering sex differences in molecular and immunological mechanisms underlying RA onset, which is significant to enhance our understanding of sex-specific RA risk factors and the development of intervention strategies personalized by sex.

## Supplementary Information

Supplementary Material 1

Supplementary Material 2

## Data Availability

Data generated from this study may be available from the corresponding author upon reasonable request.
