# Supplementary material for: Sex differences in disease severity and immune responses in murine and human inflammatory arthritis
Source: Biol Sex Differ. 2026 Feb 7;17:47. doi: 10.1186/s13293-026-00840-w (PMC12977487; doi:10.1186/s13293-026-00840-w)
Supplement: Supplementary file 1 — Supplementary Material 1 [file 13293_2026_840_MOESM1_ESM.docx]

***Supplementary Table 1. List of 44 cytokines and chemokines showing magnitude of change in***

***the serum of female and male CIA and saline control mice***

| **Sl. no** | **Cytokine/ Chemokine** | **Log_2_ fold change female (CIA-Saline)** | ***p*-value** | **Log_2_ fold change male (CIA-Saline)** | ***p*-value** | **Log_2_ fold change (Female-Male)** | ***p*-value** |
| --- | --- | --- | --- | --- | --- | --- | --- |
| 1 | Eotaxin | -0.09 | 0.7791 | 0.13 | 0.2717 | -0.22 | 0.0885 |
| 2 | G-CSF | 1.20 | 0.0076 | 1.42 | 0.0075 | -0.21 | 0.6212 |
| 3 | GM-CSF | -1.64 | 0.2379 | 1.47 | 0.2671 | -3.11 | 0.0576 |
| 4 | IFNγ | 1.04 | 0.3213 | 2.77 | 0.0058 | -1.73 | 0.0846 |
| 5 | IL-1α | 1.01 | 0.0024 | 1.96 | 0.0042 | -0.95 | 0.0030 |
| 6 | IL-1β | 0.99 | 0.0029 | 1.06 | 0.0034 | -0.07 | 0.7423 |
| 7 | IL-2 | -1.74 | 0.1220 | -0.63 | 0.6754 | -1.11 | 0.4087 |
| 8 | IL-3 | -0.43 | 0.4039 | -0.15 | 0.7653 | -0.28 | 0.5466 |
| 9 | IL-4 | 0.82 | 0.3963 | 0.46 | 0.4053 | 0.36 | 0.6968 |
| 10 | IL-5 | -0.16 | 0.6981 | 0.74 | 0.0011 | -0.90 | 0.0138 |
| 11 | IL-6 | 4.07 | 0.0005 | 5.26 | 0.0000 | -1.19 | 0.0777 |
| 12 | IL-7 | -1.17 | 0.6450 | 1.42 | 0.2088 | -2.59 | 0.1145 |
| 13 | IL-9 | -0.09 | 0.7890 | -0.31 | 0.5853 | 0.22 | 0.6561 |
| 14 | IL-10 | 1.79 | 0.1365 | 1.47 | 0.1252 | 0.32 | 0.7401 |
| 15 | IL-12p40 | -1.05 | 0.1080 | -0.87 | 0.6327 | -0.17 | 0.9092 |
| 16 | IL-12p70 | 0.52 | 0.7943 | -2.27 | 0.1670 | 2.79 | 0.1708 |
| 17 | IL-13 | -0.08 | 0.8279 | 0.45 | 0.0364 | -0.53 | 0.1389 |
| 18 | IL-15 | -0.96 | 0.4193 | 0.33 | 0.5154 | -1.29 | 0.0675 |
| 19 | IL-17 | -0.86 | 0.0553 | -1.89 | 0.0056 | 1.03 | 0.0806 |
| 20 | IP-10 | 2.17 | 0.0000 | 2.26 | 0.0000 | -0.08 | 0.6171 |
| 21 | KC | 0.73 | 0.3952 | 1.53 | 0.0037 | -0.80 | 0.2156 |
| 22 | LIF | -1.22 | 0.4834 | 0.68 | 0.4762 | -1.90 | 0.0783 |
| 23 | LIX | -0.47 | 0.4610 | 0.11 | 0.2532 | -0.58 | 0.3689 |
| 24 | M-CSF | -1.70 | 0.0207 | -3.54 | 0.0097 | 1.83 | 0.1012 |
| 25 | MCP-1 | 0.87 | 0.0025 | 2.17 | 0.0241 | -1.30 | 0.0003 |
| 26 | MIG | 0.98 | 0.0027 | 1.54 | 0.0003 | -0.56 | 0.0119 |
| 27 | MIP-1α | 1.77 | 0.0002 | 2.51 | 0.0000 | -0.75 | 0.0196 |
| 28 | MIP-1β | 1.06 | 0.0004 | 3.42 | 0.0729 | -2.36 | 0.0003 |
| 29 | MIP-2 | 1.55 | 0.2654 | 1.14 | 0.4670 | 0.41 | 0.4279 |
| 30 | RANTES | 0.57 | 0.0809 | 1.49 | 0.0005 | -0.92 | 0.0070 |
| 31 | TNFα | -0.33 | 0.1442 | 0.22 | 0.4308 | -0.55 | 0.0490 |
| 32 | VEGF | -0.88 | 0.0007 | 0.05 | 0.8963 | -0.93 | 0.0014 |
| 33 | 6Ckine/Exodus | -9.75 | 0.0162 | 0.86 | 0.0044 | -10.61 | 0.0000 |
| 34 | EPO | 0.87 | 0.0235 | 1.41 | 0.0113 | -0.54 | 0.1888 |
| 35 | Fractalkine | -0.77 | 0.0122 | -0.48 | 0.0080 | -0.29 | 0.2089 |
| 36 | IFNβ-1 | 0.96 | 0.1231 | 1.61 | 0.0029 | -0.64 | 0.2649 |
| 37 | IL-11 | 1.18 | 0.0757 | 2.38 | 0.0018 | -1.20 | 0.0013 |
| 38 | IL-16 | 0.58 | 0.2136 | 2.10 | 0.0245 | -1.52 | 0.0076 |
| 39 | IL-20 | -0.65 | 0.0396 | 2.08 | 0.0529 | -2.73 | 0.0000 |
| 40 | MCP-5 | 1.12 | 0.0188 | 1.27 | 0.0022 | -0.15 | 0.5363 |
| 41 | MDC | -0.32 | 0.3759 | 0.49 | 0.1364 | -0.81 | 0.0576 |
| 42 | MIP-3α | 0.58 | 0.1788 | 0.04 | 0.8817 | 0.53 | 0.1614 |
| 43 | MIP-3β | 1.40 | 0.0267 | 1.21 | 0.0033 | 0.19 | 0.6932 |
| 44 | TARC | 0.02 | 0.9552 | 0.05 | 0.7953 | -0.03 | 0.9209 |

***Supplementary Table 1.*** List of serum cytokines and chemokines representing the Log_2_ fold-change and corresponding *p*-values from sex-disaggregated (CIA-saline) and sex-based comparison (Female-Male) data analysis. The *p*-values were calculated using unpaired t-test.

***Supplementary Table 2. List of 44 cytokines and chemokines showing magnitude of change in the joint tissue of female and male CIA and saline control mice***

| **Sl. no** | **Cytokine/ Chemokine** | **Log_2_ fold change female (CIA-Saline)** | ***p*-value** | **Log_2_ fold change male (CIA-Saline)** | ***p*-value** | **Log_2_ fold change (Female-Male)** | ***p*-value** |
| --- | --- | --- | --- | --- | --- | --- | --- |
| 1 | Eotaxin | 4.20 | 0.002 | 2.84 | 0.00089 | 1.36 | 0.134 |
| 2 | G-CSF | 6.41 | 0.008 | 7.62 | 0.00002 | -1.20 | 0.440 |
| 3 | GM-CSF | 0.99 | 0.040 | 0.79 | 0.07296 | 0.20 | 0.629 |
| 4 | IFNγ | 2.33 | 0.004 | 1.86 | 0.00104 | 0.47 | 0.390 |
| 5 | IL-1α | 1.84 | 0.002 | 0.87 | 0.04249 | 0.97 | 0.034 |
| 6 | IL-1β | 2.45 | 0.006 | 1.65 | 0.00075 | 0.80 | 0.216 |
| 7 | IL-2 | 1.57 | 0.000 | 0.67 | 0.13006 | 0.89 | 0.027 |
| 8 | IL-3 | 1.40 | 0.017 | 1.03 | 0.01863 | 0.37 | 0.454 |
| 9 | IL-4 | 1.51 | 0.000 | 0.51 | 0.15181 | 1.00 | 0.002 |
| 10 | IL-5 | 1.40 | 0.019 | 0.82 | 0.02145 | 0.59 | 0.222 |
| 11 | IL-6 | 5.32 | 0.021 | 6.61 | 0.00000 | -1.29 | 0.437 |
| 12 | IL-7 | 1.12 | 0.005 | -0.14 | 0.67907 | 1.26 | 0.004 |
| 13 | IL-9 | 1.69 | 0.001 | 0.93 | 0.01642 | 0.76 | 0.032 |
| 14 | IL-10 | 2.03 | 0.001 | 0.61 | 0.10453 | 1.42 | 0.003 |
| 15 | IL-12p40 | 2.24 | 0.042 | 0.00 | 0.99929 | 2.24 | 0.056 |
| 16 | IL-12p70 | 2.90 | 0.003 | 1.75 | 0.00089 | 1.14 | 0.057 |
| 17 | IL-13 | 1.35 | 0.006 | 0.83 | 0.00854 | 0.52 | 0.151 |
| 18 | IL-15 | 1.75 | 0.016 | 0.60 | 0.25691 | 1.15 | 0.073 |
| 19 | IL-17 | 1.31 | 0.010 | 0.55 | 0.15430 | 0.76 | 0.077 |
| 20 | IP-10 | 3.30 | 0.002 | 2.78 | 0.00040 | 0.51 | 0.410 |
| 21 | KC | 4.71 | 0.017 | 5.77 | 0.00011 | -1.06 | 0.456 |
| 22 | LIF | 4.43 | 0.021 | 5.52 | 0.00003 | -1.09 | 0.444 |
| 23 | LIX | 2.72 | 0.005 | 1.82 | 0.01834 | 0.90 | 0.181 |
| 24 | M-CSF | 2.53 | 0.006 | 1.94 | 0.00065 | 0.59 | 0.361 |
| 25 | MCP-1 | 3.14 | 0.053 | 3.82 | 0.00034 | -0.68 | 0.611 |
| 26 | MIG | 6.64 | 0.000 | 5.66 | 0.00001 | 0.98 | 0.198 |
| 27 | MIP-1α | 2.83 | 0.025 | 3.04 | 0.00014 | -0.20 | 0.826 |
| 28 | MIP-1β | 4.21 | 0.005 | 3.93 | 0.00008 | 0.27 | 0.782 |
| 29 | MIP-2 | 5.19 | 0.008 | 4.72 | 0.00065 | 0.46 | 0.740 |
| 30 | RANTES | 2.33 | 0.000 | 1.14 | 0.02542 | 1.20 | 0.018 |
| 31 | TNFα | 2.00 | 0.006 | 1.32 | 0.00343 | 0.68 | 0.216 |
| 32 | VEGF | 3.35 | 0.002 | 2.67 | 0.00473 | 0.68 | 0.333 |
| 33 | Fractalkine | 3.14 | 0.008 | 1.40 | 0.04047 | 1.74 | 0.074 |
| 34 | IFNβ-1 | 3.99 | 0.001 | 4.50 | 0.00019 | -0.51 | 0.468 |
| 35 | IL-11 | 5.94 | 0.009 | 6.84 | 0.00009 | -0.90 | 0.542 |
| 36 | IL-16 | 2.79 | 0.065 | 3.32 | 0.00001 | -0.54 | 0.661 |
| 37 | IL-20 | 2.85 | 0.026 | 1.99 | 0.05568 | 0.86 | 0.405 |
| 38 | MCP-5 | 6.30 | 0.003 | 6.31 | 0.00007 | 0.00 | 0.999 |
| 39 | MDC | 2.91 | 0.005 | 3.51 | 0.00001 | -0.61 | 0.347 |
| 40 | MIP-3α | 1.15 | 0.004 | 0.51 | 0.04401 | 0.64 | 0.040 |
| 41 | MIP-3β | 5.07 | 0.000 | 4.60 | 0.00003 | 0.46 | 0.447 |
| 42 | TARC | 0.93 | 0.176 | 1.26 | 0.04785 | -0.32 | 0.585 |

**Supplementary Table 2**: List of cytokines and chemokines in the joints demonstrating the Log_2_ fold-change and corresponding *p*-values for sex-disaggregated (CIA-saline) and sex-based comparison (Female-Male) data analysis. The *p*-values were calculated using unpaired t-test.

***Supplementary Table 3. Baseline characteristics of patients with RA split by sex.***

|  | **Female (n = 73)** | **Male (n=19)** |
| --- | --- | --- |
| **Age** | 43.9 (13.9) | 43.6 (13.3) |
| **BMI** | 31 (8.2) | 26 (5.2) |
| **ACPA+** | 84.50% | 89.50% |
| **RF+** | 81.90% | 84.20% |
| **RF/ACPA+** | 76.40% | 78.90% |
| **CRP** | 11.8 (15.4) | 16.0 (17.0) |
| **DAS28** | 3.4 (1.3) | 3.9 (0.9) |
| **Prednisone use** | 6 (8.2%) | 2 (10.5%) |

**Supplementary Table 3.** Baseline characteristics of RA patients. Abbreviations: BMI, body mass index; ACPA+, anti-citrullinated protein antibody positivity; RF+, rheumatoid factor positivity; CRP, C-reactive protein; DAS28, Disease Activity Score 28.
