# Supplementary material for: Sex differences in disease severity and immune responses in murine and human inflammatory arthritis
Source: Biol Sex Differ. 2026 Feb 7;17:47. doi: 10.1186/s13293-026-00840-w (PMC12977487; doi:10.1186/s13293-026-00840-w)
Supplement: Supplementary file 2 — Supplementary Material 2 [file 13293_2026_840_MOESM2_ESM.docx]

**Supplementary Figures**


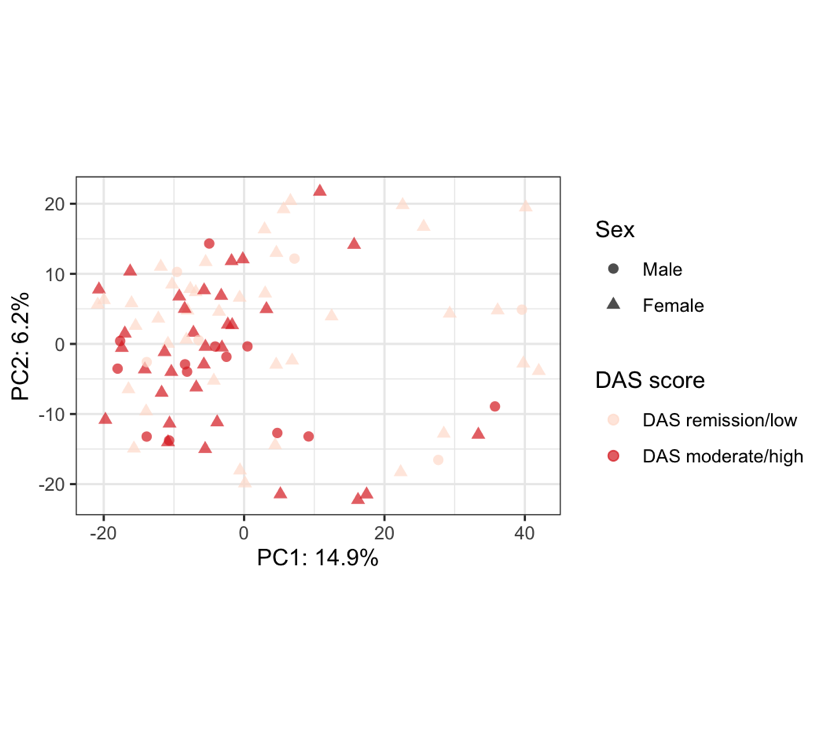

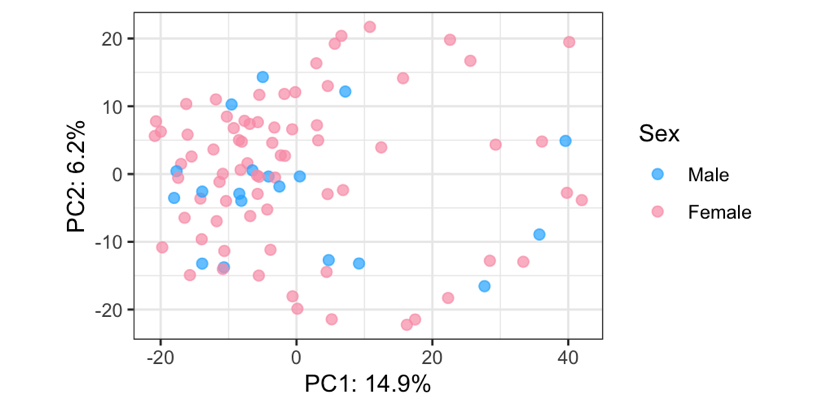
**A B**

***Supplementary Figure 1:*** Principal component analysis (PCA) of the serum proteome measured by SomaScan coloured by **(A)** sex or **(B)** disease activity measured by DAS28 score using a binary cut-off of remission/low disease activity or moderate/high disease activity.


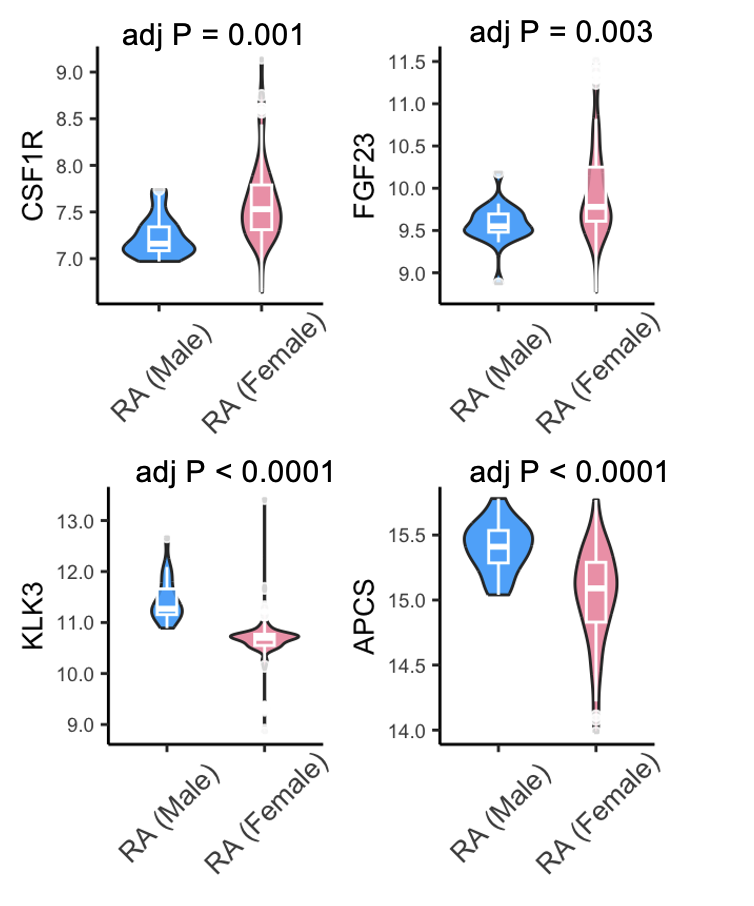


***Supplementary Figure 2****:* Differentially expressed proteins in RA between each sex measured by SomaScan. Differences were calculated by pairwise t-test corrected for multiple testing by Benjamini-Hochberg (adj P = adjusted P value).


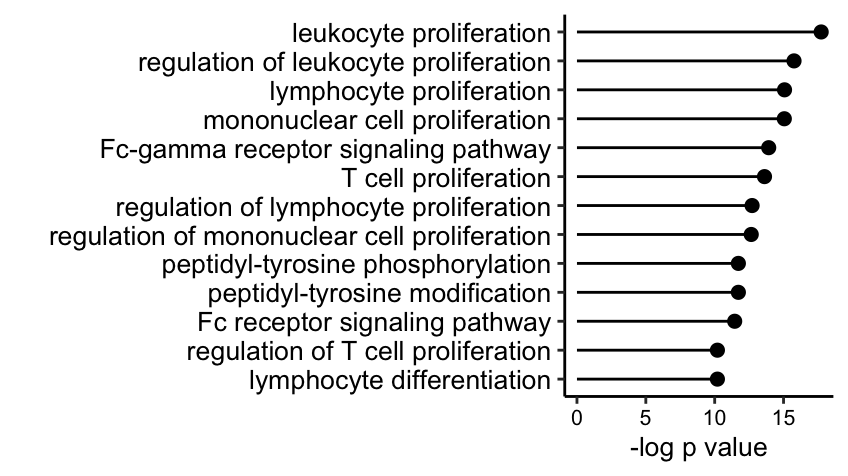


***Supplementary Figure 3:*** Upregulated Gene Ontology (GO) pathways in female RA patients using male RA patients as a reference.


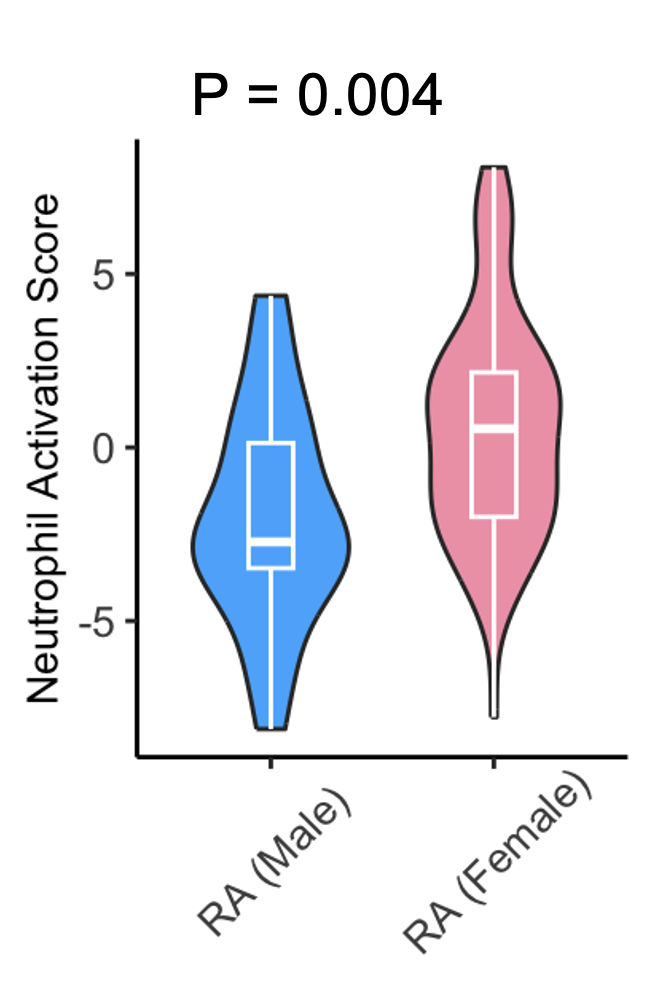


***Supplementary Figure 4:*** Neutrophil activation score is higher in female than male RA patients. The neutrophil activation score was derived by summating the Gene Ontology (GO) enrichment proteins for neutrophil activation. Differences were calculated by student t-test.
